# Development of Poly(lactic acid)/Chitosan Fibers Loaded with Essential Oil for Antimicrobial Applications

**DOI:** 10.3390/nano7070194

**Published:** 2017-07-24

**Authors:** Yaowen Liu, Shuyao Wang, Rong Zhang, Wenting Lan, Wen Qin

**Affiliations:** 1 College of Food Science, Sichuan Agricultural University, Yaan 625014, China; shuyaow@126.com (S.W.); 18227593325@163.com (R.Z.); 18227593253@163.com (W.L.); qinwen1967@yahoo.com.cn (W.Q.); 2 School of Materials Science and Engineering, Southwest Jiaotong University, Chengdu 610031, China

**Keywords:** cinnamon essential oil, chitosan, poly(lactic acid), antibacterial fibers

## Abstract

Cinnamon essential oil (CEO) was successfully encapsulated into chitosan (CS) nanoparticles at different loading amounts (1%, 1.5%, 2%, and 2.5% *v*/*v*) using oil-in-water (o/w) emulsion and ionic-gelation methods. In order to form active packaging, poly(lactic acid) (PLA) was used to fabricate PLA/CS-CEO composite fibers using a simple electrospinning method. The shape, size, zeta potential, and encapsulation efficacy of the CS-CEO nanoparticles were investigated. The composition, morphology, and release behavior of the composite fibers were investigated. PLA/CS-CEO-1.5 showed good stability and favorable sustained release of CEO, resulting in improved antimicrobial activity compared to the other blends. The PLA/CS-CEO fibers showed high long-term inactivation rates against *Escherichia coli* and *Staphylococcus aureus* due to the sustained release of CEO, indicating that the developed PLA/CS-CEO fibers have great potential for active food packaging applications.

## 1. Introduction

Currently, many new active packaging materials are receiving increasing attention in the food packaging industry. Active packaging can hinder the growth of microorganisms on the food surface, improve nutritional and sensory qualities of food, extend the shelf-life of certain foods, and decrease the environment impact of the packaging. Active packaging technologies can be based on synthetic or natural materials, and some contain active constituents, such as antioxidants, antimicrobials, vitamins, flavors, or colorants. Cinnamon essential oil (CEO) is a natural antimicrobial substance which is being investigated for food packaging as a replacement for synthetic chemicals due to consumer concerns over food safety. In particular, CEO has low toxicity, low environmental impact, and high antibacterial and antioxidant activity [1]. However, the application of CEO in active packaging is challenging due to its hydrophobicity, high volatility, and susceptibility to degradation from exposure to oxygen, heat, and light [2]. In order to overcome these challenges, many studies have shown that the most effective method is to encapsulate the essential oil in a carrier to extend its applicability. Encapsulation protects CEO from hostile environmental conditions, thereby extending the shelf life of the product and enabling controlled release of the active compound [3]. Many polymers, such as liposomes, sodium alginate, and chitosan, have also been widely used to encapsulate and increase the stability and bioactivity of essential oils [4]. 

Chitosan (CS) is a cationic natural polymer derived by the deacetylation of chitin, the major component of the shells of crustaceans. It has a great potential to be used as active packaging due to its antibacterial and antifungal properties. Chitosan has been loaded with cinnamon essential oil, *Eucalyptus staigeriana* essential oil, oregano essential oil, and limonene essential oil with the intention of enhancing their stability under given environmental or processing conditions and retaining their antimicrobial activity [5]. Ghaderi-Ghahfarokhi et al. produced CEO-incorporated CS nanoparticles by an emulsion-gelation method. They found that the encapsulated CEO was useful for preventing lipid oxidation and microbial spoilage in beef patties [6]. Feyzioglu et al. incorporated different concentrations of Summer savory (*Satureja hortensis* L.) essential oil into CS nanoparticles. They showed that the incorporation of the this essential oil increased thermal stability and water-holding capacity of the CS and demonstrated high bioactive properties for future active packaging applications [7]. Sotelo-Boyas et al. compared the antibacterial activity of CS nanoparticles and nanocapsules incorporated with lime essential oil prepared by nanoprecipitation and nanoencapsulation methods. They observed the highest antibacterial activity against *S. dysenteriae* for the composite particles synthesized by nanoprecipitation [8]. EO/CS nanoparticles obtained using such methods demonstrated some antibacterial activity, but do not meet all the requirements for a good packaging material. Zhang et al. used oil/water emulsions and ionic gelation methods to encapsulate clover, cinnamon bark, and lemongrass oils in CS beads, where the mechanical and antibacterial properties of composite films were enhanced by incorporating cellulose films [1]. Kashiri et al. produced zein films with different concentrations of *Zataria multiflora* Boiss. essential oil, which were proposed as an active food packaging for pasteurized cow’s milk [9].

Among those encapsulation methods, electrospinning is a maturing technique that facilitates the production of polymeric fibers incorporated with EO-loaded particles. In additon, pure CS is difficult to electrosping due to the poor entanglement of the chains. Hence, CS is often blended with synthetic polymers such as PVA, poly(caprolactone), and poly(lactic acid) (PLA) to enhance the spinability of the CS polymer and improve the mechanical properties of the resulting fibers [10]. Wen et al. reported electrospun polyvinyl alcohol/CEO/b-cyclodextrin (PVA/CEO/b-CD), which exhibited excellent antimicrobial activity against *Escherichia coli* (*E. coli*) and *Staphylococcus aureus* (*S. aureus*) demonstrated an effective extension of the shelf-life of packaged strawberries [11]. They chose PLA as the polymer backbone of the fibers, because PLA is a bio-plastic which has been approved by the U.S. Food and Drug Administration (FDA) and is widely used in material that comes in contact with food [12]. Wen et al. produced PLA/CS-CEO composite fibers and demonstrated that PLA/CEO/b-CD fiber effectively prolonged the shelf life of pork, indicating that such fibers have potential applications in active food packaging [13].

In this study, we investigated the effect of different CEO contents (1%, 1.5%, 2%, and 2.5% *v*/*v*) on the encapsulation efficiency, particle size, and antibacterial properties of CS nanoparticles incorporated with this essential oil. In addition, the structural and morphological properties of PLA/CS fibers with different CEO loadings were evaluated. This biodegradable PLA/CS/CEO fibrous mat is proposed to have excellent antimicrobial activity, making it a promising active food packaging material.

## 2. Results and Discussion

### 2.1. Physicochemical Properties of CEO-Loaded CS Particles

**Figure 1:**
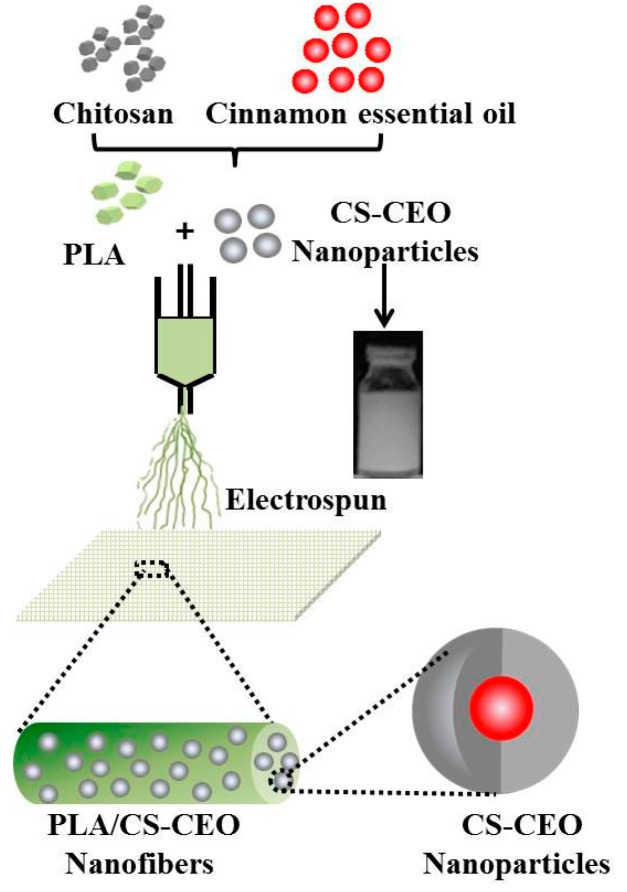
Schematic diagram of the steps for preparing the poly(lactic acid)/chitosan-cinnamon essential oil (PLA/CS-CEO) fibers used in the experiments.

The steps for preparing the PLA/CS-CEO fibers are shown in Figure 1. Briefly, The PLA was dried overnight at 60 °C in a vacuum oven, and then dissolved at 25 wt % in 80:20 (*v*/*v*) trifluoroacetic acid:dichloromethane, stirred for 12 h at room temperature, CS was dissolved in an aqueous acetic acid solution. Then, an ethanolic CEO solution was slowly added to the CS solution under continuous vigorous stirring to obtain a volume ratio of CS/CEO of 1%, 1.5%, 2%, and 2.5%. So the blended PLA/CS-CEO electrospun fibers were denoted as PLA/CS-CEO-1, PLA/CS-CEO-1.5, PLA/CS-CEO-2, and PLA/CS-CEO-2.5, where the values refer to the relative CEO concentration.

**Figure 2:**
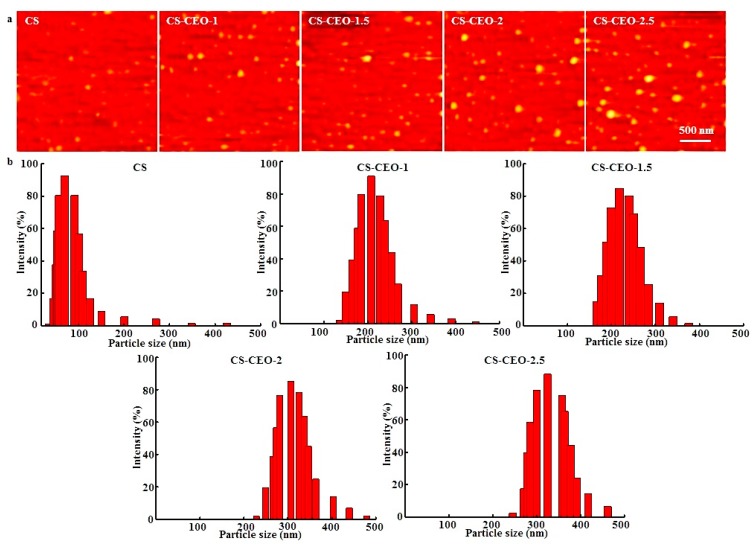
Characterization of CS-CEO nanoparticles. (**a**) AFM image and (**b**) particle size distribution of the CS-CEO particles with different CEO concentrations.

Atomic force microscopy (AFM) was used to analyze the morphology of the CS particles loaded with CEO. As can be seen in Figure 2a, the CS particles loaded with different amounts of CEO all had a spherical surface morphology, where some aggregation was observed. The mean particle diameter was found to be between 100 and 300 nm. The observed aggregation may have been a result of poor stability of the CS particles during the fabrication process. The loaded CS particles had a similar morphology to that of the bare CS nanoparticle. The average size of the CS particles without CEO loading was 79.42 ± 3.52 nm, while those loaded with CEO were significantly larger (*p* < 0.05), where the average diameters of the nanoparticles ranged from 105.27 ± 6.41 nm to 237.63 ± 12.56 nm. The size of the nanoparticles increased slightly after incorporation of the CEO. CS-CEO-2.5 had the largest particle size (*p* < 0.05) while the smallest CEO-loaded CS particles (*p* < 0.05) were for the CS-CEO-0.5 sample. The different nanoparticle sizes may have resulted from the different CS concentrations in the composite solutions [14]. In addition, it was also observed that higher relative amounts of adsorbed essential oil resulted in larger particle sizes [15]. Our findings were similar to those presented in other studies which reported that higher CS concentrations resulted in an increase in particles size [16]. 

Similarly, dynamic light scattering (DLS) measurements were used to investigate the hydrodynamic diameter of the particles, as shown in Figure 2b. CS had a unimodal distribution, with a range of 50–150 nm, while the CS-CEO samples showed a bimodal particle size distribution with a range of 150–450 nm. The results also indicated that a higher content of CEO produced larger CS-CEO particles. In general, the size of the nanoparticles tend to depend on the preparation method and experimental conditions of CS. Woranuch et al. obtained CS-CEO nanoparticles synthesized by an oil-in-water emulsion followed by ionic gelation, with particle size ranging from 80 to 100 nm [14]. Lozano et al. obtained docetaxel-loaded CS with an average size of 151.2 ± 1.5 nm, prepared by oxidative degradation of medium molecular weight CS using the solvent displacement technique [17], similar method to the one used here. Further studies are necessary to elucidate the precise mechanisms causing the formation of different sized CS nanoparticles loaded with EOs.

Table 1 summarizes the zeta potential (ZP) of different CS-CEO nanoparticles. The ZP of the CS nanoparticles was expected to be the highest. One possible reason is the formation of CS nanoparticles depends on the interaction of positively-charged chitosan and negatively-charged tripolyphosphate (TPP) [18]. All of the chitosan NPs loaded with different concentrations of CEO had negative ZP values varying from −8.6 ± 0.5 mV to −22.4 ± 1.8 mV. Significant (*p* < 0.05) decreases in ZP values were observed with increasing CEO concentration [19]. This is probably due to the presence of –NH^3+^ ions, which were responsible for interactions between the CS and CEO [20]. The zeta potentials of all CS-CEO nanoparticles were around −20 mV, indicating their good stability [7]. The efficiency of CS/CEO nanoparticles formation was significantly lower than that of the CS nanoparticles, which could be related to the hydrophilic groups of CS having lower affinity with the oil system, leading to CEO leaking out of the nanoparticles, resulting in low effective encapsulation during nanoparticle formation. The EE (%) values of CS nanoparticles loaded with CEO-1, CEO-1.5, CEO-2, and CEO-2.5 were 64.83%, 65.44%, 65.01%, and 64.74%, respectively, showed no significant difference (*p* > 0.05) for the different CEO concentrations. This suggests that CS is very well suited for encapsulation of EOs. These findings were in agreement with those of previous studies [8]. The EE% decreased with increasing CEO concentration from 55.64 ± 11.57% to 47.67 ± 8.94% (*p* > 0.05). This critical CEO concentration and EE can be attributed to inadequate CS concentration for further encapsulation of the essential oil [7]. However, these results suggest that CS nanoparticles are very well suited for the encapsulation of essential oils.

### 2.2. Morphology of PLA/CS-CEO Fiber Mats

**Figure 3:**
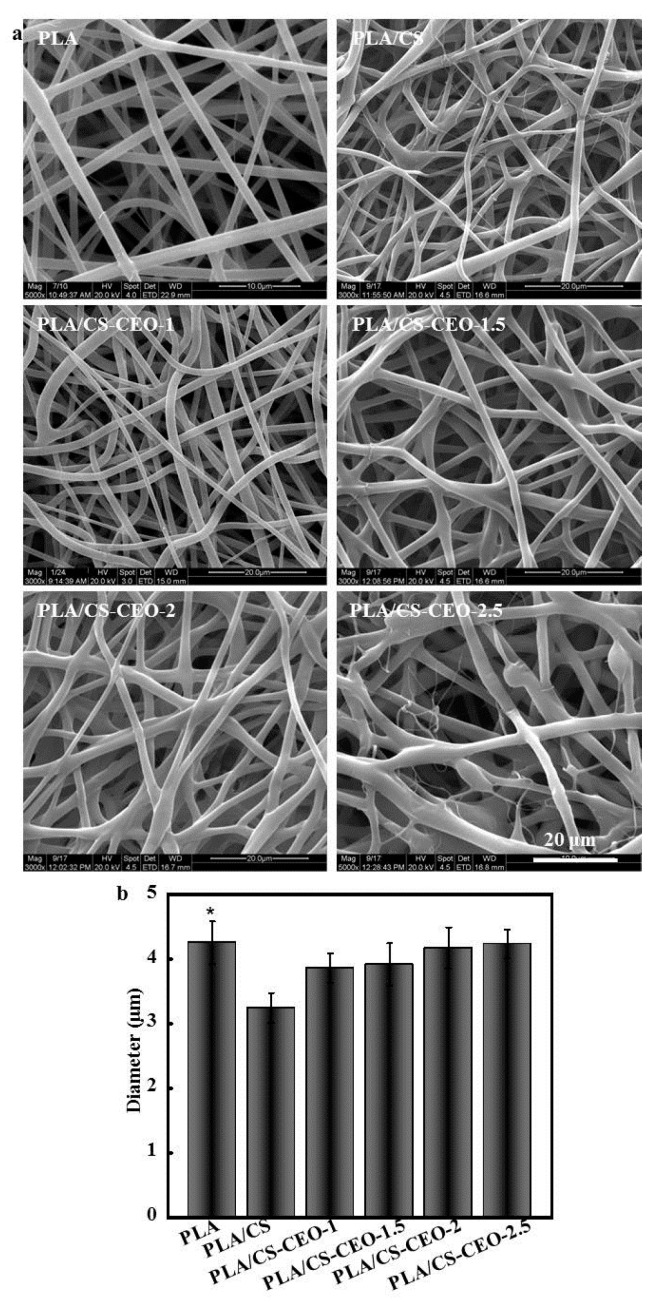
(**a**) Scanning electron micrographs of electrospun PLA/CS-CEO fibers; and (**b**) averaged diameter of PLA/CS-CEO fibers. (*n* = 5, * means *p* < 0.05).

As displayed in Figure 3a, PLA/CS-CEO fiber mats were successfully produced by electrospinning. The composites of PLA and CS encapsulated with different concentrations of CEO showed a smooth surface structure; the fibers were cylindrical with smooth surfaces (without beads) and no visible separation of the particles from the fiber matrix. Such a morphology confirmed that the CS-CEO particles were successful encapsulated into the PLA fibers. It is interesting to note that when the concentration of CS-CEO was increased to ≥2.5%, the fibers had a smaller diameter and the simultaneous formation of beads was observed (Figure 3b). In general, a low viscosity of a polymer solution can result in an increase in the surface tension of the solvent, which favors the formation of beads [21].

The average diameter of the PLA/CS-CEO fibers was much smaller than that of pure PLA fibers (4.34 ± 0.56 µm). This is attributed to the different ionic conductivities of CS. The CS molecule contains amino groups, which result in a high ionic conductivity when dissolved in an acidic solvent. This increases the charge density of the polymer jet during the electrospinning process; therefore, the CS solution could be stretched into thinner fibers with smaller diameters compared to the PLA. It was found that the average fiber diameter increased with increasing CS-CEO content. The mean diameters of PLA/CS-CEO fibers ranged from 3.87 ± 0.45 to 4.32 ± 0.53 µm. Variations in the mean fiber diameter were also attributed to changes in the viscosity of the solutions [22]. An increase in the concentration of polymer solution resulted in an increase in viscosity and, hence, an increase in the mean fiber diameter [23].

### 2.3. Fourier Transform Infrared Spectroscopy (FTIR)

**Figure 4:**
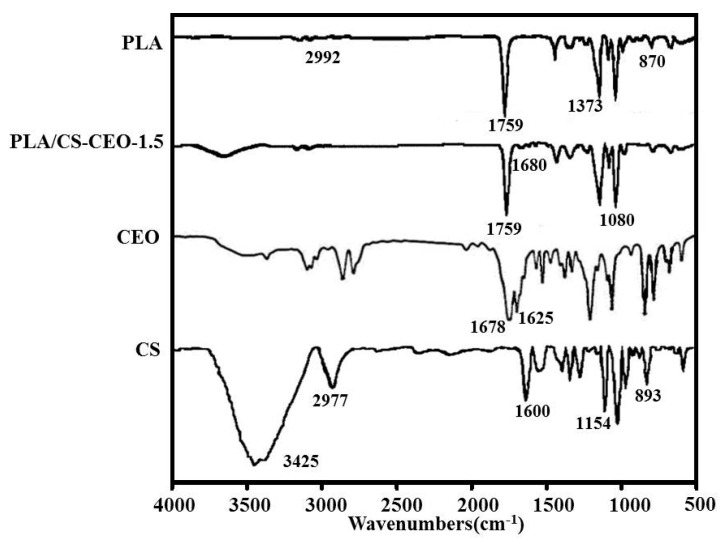
FT-IR spectrum of PLA, CEO, CS, and typical PLA/CS-CEO-1.5 fibers.

Figure 4 shows the FTIR spectra of PLA, CEO, CS, and typical PLA/CS-CEO-1.5 samples. The spectrum of CEO showed characteristic bands at 1625 and 1678 cm^−1^ corresponding to skeletal vibrations relating to C–C stretching in the benzene ring and the carbonyl group (C=O). Additionally, the peaks in the region from 1500 to 1000 cm^−1^ and from 900 to 750 cm^−1^ in the spectrum of pure CEO were due to the bending of the aromatic ring C–H bonds [11]. In the CS spectrum, characteristic absorption bands were observed at 1154 and 893 cm^−1^ (saccharide groups), 1535 cm^−1^ (N–H bending), 1600 cm^−1^ (amide I stretching), 1647 cm^−1^ (amide II bending), 2977 cm^−1^ (C–H stretching), and 3425 cm^−1^ (N–H stretching), along with a broad peak between 3400 and 3700 cm^−1^ that corresponds to O–H stretching [24]. In the PLA spectrum, characteristic peaks were observed at 2992 cm^−1^ (–CH asymmetric stretching), 1759 cm^−1^ (–C–O stretching), 1454 cm^−1^ (–CH bending in –CH_3_), 1373 cm^−1^ (–CH_3_ symmetric deformation), 1185 and 1089 cm^−1^ (–C–O stretching), and 870 cm^−1^ (–C–C stretching), as reported in the literature [25]. In the case of PLA/CS-CEO fibers, characteristic peaks of PLA and CS were observed. In general, the intensity of most of the CEO peaks depended on the concentration of the encapsulated CEO. This indicates effective encapsulation of the CEO in the CS and their good interaction. The peak at 1080 cm^−1^ indicates the presence of amino groups in CS [26]. In addition, the characteristic peaks of the CEO at 1625 cm^−1^ and 1678 cm^−1^ shifted to 1628 cm^−1^ and 1680 cm^−1^, respectively, in the PLA/CS-CEO-1.5 spectrum, while the CEO peaks were significantly weaker. This suggests that there existed host–guest interactions between the CEO and CS; a similar phenomenon was also observed in previous studies [11].

### 2.4. Surface Hydrophilicity of PLA/CS-CEO Fibers

**Figure 5:**
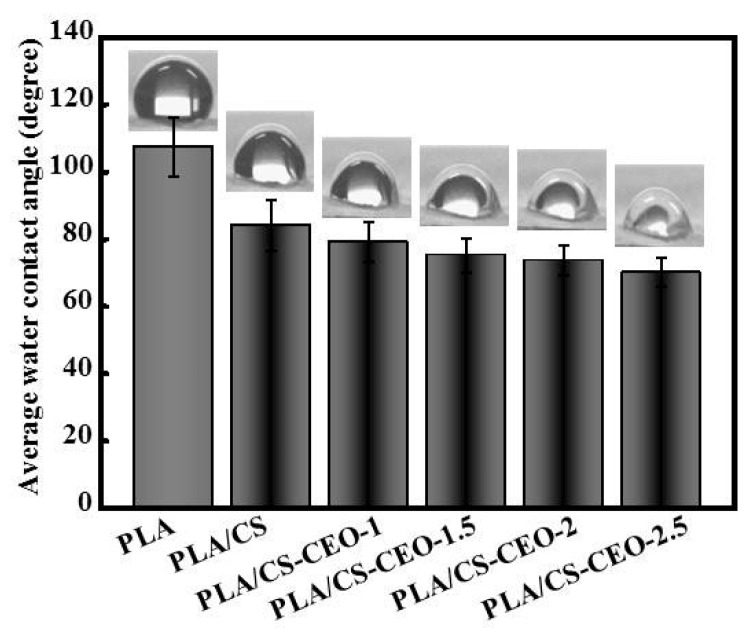
Photographs of water droplets from which average water contact angles of PLA/CS-CEO electrospun fibers were measured; *n* = 5.

Figure 5 shows the water contact angles on PLA/CS-CEO fibers prepared using different CS-CEO concentrations. The water contact angle of pure PLA was 114.5 ± 12.4°, indicating that the PLA fibers were hydrophobic polymer. The water contact angles on PLA/CS-CEO-1, PLA/CS-CEO-1.5, PLA/CS-CEO-2, and PLA/CS-CEO-2.5 fibers were 82.5 ± 10.6°, 80.6 ± 9.8°, 78.8 ± 8.4°, and 76.5 ± 8.1°, respectively. PLA/CS-CEO fibers showed hydrophilicity, attributed to the presence of CS; a higher CS content hindered CEO diffusion from the inner core to the external region of the fibrous mats. It is clear that the addition of CS/CEO significantly decreased the water contact angle, and higher concentrations of CS/CEO resulted in PLA/CS-CEO fibers with increasingly hydrophilic behavior. This is probably due to the presence of larger amounts of –OH groups on the surface of the composite fiber [11].

### 2.5. Release Characteristics of PLA/CS-CEO Fiber Mats

**Figure 6:**
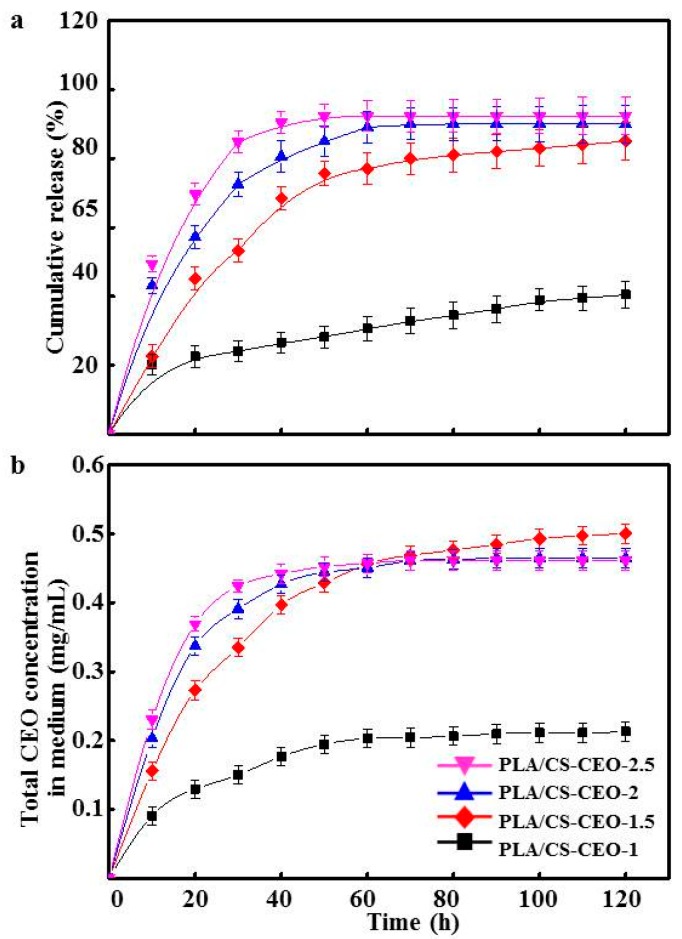
(**a**) Cumulative release of CEO from the PLA/CS-CEO fibers; and (**b**) total CEO concentration in medium from PLA/CS-CEO electrospun fibers.

The release profiles of CEO were conducted at physiological temperature (37 °C) at times relevant to antibacterial activity tests. Figure 6a,b shows the in vitro release profile of CEO from PLA/CS-CEO fibers can be described as a two-step biphasic process, i.e., an initial burst release followed by subsequent slower release. All PLA/CS-CEO fibers correspond to the rapid release phase (burst effect) after 10 h. The burst effect was reported to be the result of high oil content on the surface of the particles and oil entrapped near the surface; the CEO loosely bound near the surface of fibers [27]. For the PLA/CS-CEO-1, the value of the diffusional exponent n was 0.24105, considering Peppas’ equation explaining the non-Fickian transport mechanism, this exponent shows that the release rate depends simultaneously on the swelling and the presence of CEO at the surface or in the exterior layer of the fibers. After 10 h, the release of CEO from PLA/CS-CEO-1.5, PLA/CS-CEO-2, and PLA/CS-CEO-2.5 showed a gradual increase in cumulative release. This may have been the result of CEO trapped in the inner core of the fiber matrix diffusing to the surface. Moreover, the cumulative release percentage and total CEO concentration in medium increased with increasing CEO contents. In contrast, the cumulative amount and CEO concentration in medium released from PLA/CS-CEO-2.5 fibers was statistically higher than PLA/CS-CEO-1 fibers (*p* < 0.05) between 0 and 60 h. After 60 h, the total CEO concentration in medium released from PLA/CS-CEO-1.5 fiber is statistically higher than the CEO released from other fibers (*p* < 0.05). Overall, 75% more CEO was released from the fiber with the highest CEO concentration in medium (0.45 mg/mL), and the release started to level off indicating that equilibrium has been reached [10]. A possible reason for this is that the incorporation of CEO might destroy hydrogen bonding between CS and PLA chains [28], and the dissolution rate of the polymer near the surface is high, the amount of CEO released will be also high. In addition, the small amount of CEO released from the PLA/CS-CEO-1.5 fiber after 60 h, and shown a long-term release of CEO, which could be explained by a low degree of swelling and the rigid polymeric chains which did not permit CEO diffusion [29]. For the PLA/CS-CEO-1.5 sample, we observed values of 0.43 < *n* < 0.85, which demonstrated that the CEO release is a diffusion-swelling controlled process. This could be a result of the low degree of swelling and the presence of oil droplets at the surface or in the exterior layer of the microcapsules. These values were close to those obtained in the present study [29]. Further release of CEO also depends on the volatility of CEO, morphology, and composition of the PLA/CS-CEO fibers [10]. Hence, the results indicate that the PLA/CS-CEO-1.5 fiber is suitable for controlling the release of CEO. 

### 2.6. Antibacterial Activity of PLA/CS-CEO Fibers

**Figure 7:**
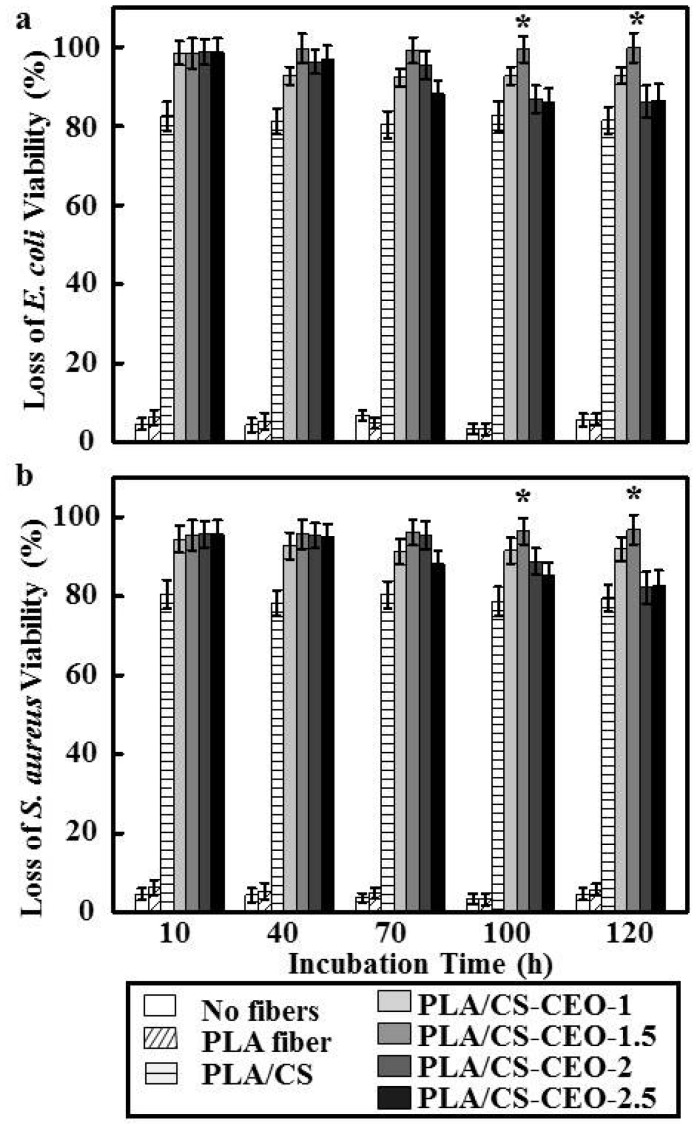
The loss of (**a**) *E. coli* and (**b**) *S. aureus* viability of incubation time for PLA, PLA/CS, and PLA/CS-CEO electrospun fibers. (*n* = 5, * means *p* < 0.05).

As mentioned above, CS and essential oils have a synergistic antibacterial effect [30]. As shown in Figure 7, pure PLA did not show any antibacterial activity, while PLA/CS-CEO fibers showed antimicrobial activity against both *E. coli* and *S. aureus* for a period of 120 h, where the antibacterial activity increased with increasing CEO content. Although PLA/CS-CEO-1 showed some inhibition of *E. coli* and *S. aureus*, the antimicrobial activity depended on the CS, CEO concentration, and time. Cinnamaldehyde, the key compound present in the essential oil, reduces the activity of enzymes causing protein denaturation and increases the microbial cell wall permeability, thereby permitting leakage of cell components, resulting in cell death [31]. However, we also found that the inhibitory effect of PLA/CS-CEO-2 and PLA/CS-CEO-2.5 fibers gradually decreased after a treatment time of 70 h, which was similar with the release studies. After 70 h, PLA/CS-CEO-2 and PLA/CS-CEO-2.5 fibers had released almost all CEO to the medium. Therefore, the final amount of CEO liberated into the medium slowed down over time. Furthermore, this indicated that the controlled release of CEO from composite films is critical for ensuring the antibacterial activity against these test strains [32]. The maximum concentration was achieved on the PLA/CS-CEO-1.5 sample, which showed complete inhibition of *E. coli* and *S. aureus* during the incubation period, and the highest antibacterial efficiency of 99.3% and 98.4%, respectively. This was possibly due to the strong interactions between CS and CEO and high crystallinity of CEO resulting in lower solubility of PLA, allowing the CEO to exert a similar antimicrobial action even when it is released more slowly [9]. It is interesting that PLA/CS-CEO were more effective against Gram-negative *E. coli* than Gram-positive *S. aureus* (Figure 6). We hypothesize that this is due to hydrophilic CS loaded with CEO passing through the outer membrane via abundant pores in proteins that serve as hydrophilic transmembrane channel in the *E. coli* [1]. However, further studies should be conducted to prove the antibacterial mechanisms of EOs released from CS particles to validate this hypothesis. 

## 3. Materials and Methods

### 3.1. Materials

We synthesized PLA (Mw = 15,000) by lactide and ring opening polymerization as previously reported [33]. The CS (Mw 8000-1, 2000; deacetylation 85%) was provided by Sinopharm Chemical Reagent Co., Ltd. (Chengdu, China). Acetic acid (>99.7% purity) and pentasodium tripolyphosphate (TPP) were purchased from Sigma-Aldrich (St. Louis, MO, USA). Methanol, acetic acid, glycerol, and Tween 80 were purchased from Merck (Darm-stadt, Germany). The cinnamon essential oil was purchased from Hengcheng Natural Flavours Co. (Jiangxi, China). Difco Luria-Bertani (LB) broth was purchased from BD Life Sciences (Franklin Lakes, NJ, USA). All other chemicals and solvents were reagent grade or higher purity and were purchased from Chengdu Kelong Reagent Co. (Chengdu, China) unless otherwise indicated.

### 3.2. Preparation of CS-CEO Nanoparticles

The CS-CEO nanoparticles were prepared following a published literature with some modifications [7]. Briefly, CS (0.5% *w*/*v*) was dissolved in 40 mL aqueous acetic acid solution (1% (*v*/*v*)) while continuously stirring under ultrasonication for 1 h. Then Tween 80 surfactant (1:1, *v*/*v*) was subsequently added to the CS solution with constant stirring at 50 °C for 1h to obtain a homogeneous solution. Then ethanolic CEO solution (20 mg/mL) was slowly added to the CS solution at a rate of 5 mL/h under continuous vigorous stirring for 50 min to obtain a volume ratio of CS:CEO with 1%, 1.5%, 2%, and 2.5%. The pH of the mixture was maintained in the range of 4.5–5. After the formation of a fine homogenization, 15 mL of TPP solution was slowly dropped into the solution under continuous stirring for 50 min. CS nanoparticles were also synthesized using the same protocol.

### 3.3. Characterization of CS-CEO Nanoparticles

CS-CEO nanoparticles were observed by atomic force microscopy (AFM; CSPM5000, Shanghai, China) after the sample-loaded silicon wafer was air-dried at room temperature. The average particle size and zeta potential were measured using a Nano-ZS laser particle analyzer (Zetasizer Nano ZS90, Malvern Co., Worcestershire, UK). The encapsulation efficacy of the CEO was estimated by comparing the amount of CEO initially added for encapsulation and the amount of CEO left in the supernatant after centrifugation. The amount of free CEO in the ethanol before encapsulation and in the collected supernatant after encapsulation and centrifugation were detected using UV/Vis spectrophotometry at 275 nm (UV-1800, Thermo Fisher Scientific, Inc., New York, NY, USA). Then, the absorbance of the supernatant was measured over the wavelength range of 250–400 nm. Maximum absorption was observed at a wavelength of 289 nm [34]. The nanoparticle forming efficiency indicated the amount of nanoparticles retrieved compared with that of polymers used for the nanoparticle preparation. The CEO loading in the PLA/CS-CEO samples was determined as follows. A typical extraction involved mixing a sample of the CS-CEO powder (20–30 mg) with distilled water (20 mL) and ethyl acetate (10 mL) in a 100 mL stoppered conical flask. The solution was then heated on a heating block at 85 °C for 20 min, with intermittent shaking. The organic phase containing the volatile compounds was decanted and the aqueous phase was extracted two times using ethyl acetate at 85 °C using the above method. The concentration of CEO in the ethyl acetate was determined by UV–VIS spectrophotometry (UV-2550, Shimadzu, Japan) using a standard CEO concentration–absorbance calibration curve [11].

### 3.4. Preparation of PLA/CS-CEO Fibers

PLA was dried at 60 °C in a vacuum oven overnight, and then dissolved at 25 wt% in 80:20 (*v*/*v*) trifluoroacetic acid:dichloromethane, stirred for 12 h at room temperature, and then mixed with targeted CEO/CS with ratios of 0%, 1%, 1.5%, 2%, and 2.5% (*v*/*v*), where the blended electrospun fibers were denoted as PLA/CS, PLA/CS-CEO-1, PLA/CS-CEO-1.5, PLA/CS-CEO-2, and PLA/CS-CEO-2.5, respectively. The solution was then loaded at 1.0 mL/h and 40–50% humidity into a circular metal capillary with 0.7 mm inner diameter, using a syringe pump (Zhejiang University Medical Instrument Company, Zhejiang, China) fitted with a 5 mL syringe. A high-voltage statitron (Tianjing High Voltage Power Supply Co., Tianjin, China) was then used to apply a voltage difference of 20 kV between the syringe nozzle and a grounded collector placed 15 cm apart. A plate-type collector covered with aluminum foil was used to collect the fiber mats. The collected fibers were vacuum-dried at room temperature for two days to completely remove residual solvent. 

### 3.5. Characterization of PLA/CS-CEO Fibers

The chemical interactions between PLA, CEO, and CS were investigated using FTIR spectroscopy (Equinox 55, Bruker Co., Ettlingen, Germany). During the FTIR measurements ATR was used for the electrospun fiber, while KBr disks were used for physical mixtures of PLA, CEO, and CS. Analysis of the FTIR spectra was performed in the mean infrared region over a wavenumber range of 4000–500 cm^−1^, with a spectral resolution of 4 cm^−1^. The signals were processed using the OPUS spectroscopic software. The fibrous mats were imaged using scanning electron microscopy (SEM; FEI Quanta 200, Eindhoven, The Netherlands) operating at accelerating voltage of 20.0 kV. The fiber diameter was determined manually using ImageJ software (version 1.46, Scion Corporation, Frederick, MD, USA) from at least 50 randomly-selected sites of three randomly-selected SEM micrographs obtained at a magnification of 2000×. The hydrophilicity was determined using a video-based optical contact angle meter (Data Physics OCA 15EC). Samples with dimensions of 4 cm × 4 cm were fixed on a glass microscope slide with double-sided tape to ensure a uniform view of the surface. The slide was then placed on the meter stage where a 5 µL drop of water was placed on the surface. The contact angle on both sides of the drop was measured using the software, and the average of ten angles was reported for each sample. An in vitro release study was performed using the dialysis method. In brief, a dialysis bag was soaked in distilled water to remove the preservatives and rinsed with phosphate-buffered saline (PBS) solution. CEO-loaded nanoparticles were redispersed in 3 mL of PBS solution and loaded into the dialysis bag with 50 mL of PBS containing 20% ethanol with pH 7.4. The use of ethanol helped to minimize fiber aggregation and release the oil more uniformly [35]. A time-dependent release study was performed over 120 h, all samples were incubated at 37 °C under gentle agitation. At defined time intervals, 3 mL of the medium was removed for quantification and replaced with fresh medium. The release was quantified as the percentage of released oil relative to the total amount of oil [36]. CEO concentration in the released solution was determined using UV–VIS spectroscopy (UV–VIS, Agilent diode array) at an absorbance of 293 nm. The kinetics of CEO release was analyzed using the parameters in Peppas’ equation [29]:

Q = *k* × *t^n^*

where Q is the cumulative percent of essential oil released at time *t*; *k* is a constant related to structural and geometric characteristics of the matrix, and *n* is the diffusional exponent that indicates the mechanism of the essential oil release. The values of n indicate the following release mechanisms: for *n* < 0.43 the dominant release mechanism is Fickian diffusion (case I transport); 0.43 < *n* < 0.85 indicates the diffusion and the swelling release mechanism (non-Fickian or anomalous transport) and *n* > 0.85 corresponds to zero order release kinetics (case II transport) [27].

### 3.6. Antibacterial Properties of PLA/CS-CEO Fibers

The antimicrobial effectiveness of the PLA/CS-CEO fibers against *E. coli* ATCC 29522 and *S. aureus* ATCC 29523 were evaluated using a modified ASTM shake flask method [10]. An electrospun mat (10 mm × 20 mm) was immersed in a test tube containing 4 mL of an isotonic solution into which 0.5 mL of the *E. coli* and *S. aureus* inoculums, adjusted to a cell concentration of 10^7^ CFU/mL, were introduced. The test tubes were shaken at 200 rpm at 37 °C and the suspension solution was sampled from the test tubes at each analysis time and serially diluted with buffered peptone water. A sample of 100 μL of these suspensions were then spread onto LB plates using the spread plate method. These plates were incubated for 24 h at 37 °C and the colony-forming units (CFU) were counted. The percent reduction of bacteria was calculated according to:

Reduction of bacteria (%) = (*B* − *A*) / *B* × 100

where *A* is the mean log_10_ density of bacteria for treated substrate and *B* represents the untreated substrate. No fiber mat and PLA fibers were used as a control sample [10].

### 3.7. Statistical Analysis

All data are reported as mean ± standard deviation (SD), and were analyzed by analysis of variance. Values of *p* less than 0.05 were considered statistically significant.

## 4. Conclusions

CS-CEO nanoparticles were used as a carrier for load CEO for active packaging materials. PLA/CS-CEO fibers were obtained by electrospinning in order to achieve the sustained release of CEO. The effect of the amount of loaded CEO on the structure and morphology of the fibers was studied using FTIR, SEM, and AFM analyses. The results indicated that incorporating CEO could improve the antibacterial properties of the PLA/CS-CEO fibers. The optimal composition was found to be PLA/CS-CEO-1.5, which showed the highest antibacterial efficiency for a long time. Hence, such PLA/CS-CEO fibers exhibited a significant potential for active food packaging and other applications where antibacterial activity is required.

## Figures and Tables

**Table 1:** The characteristics of cinnamon essential oil (CEO)-loaded chitosan (CS) nanoparticles.

Nanoparticles	Zeta Potential (mV)	Amount of Encapsulated EO (g)	Encapsulation Efficlency (%)	Nanoparticle Formation Efficiency (%)
**CS**	−8.6 ± 0.5	-	-	76.4 ± 12.2
**CS/CEO-1**	−11.2 ± 0.8	0.32 ± 0.06	55.64 ± 9.57	74.7 ± 11.8
**CS/CEO-1.5**	−13.6 ± 1.1	0.46 ± 0.07	53.64 ± 8.44	73.6 ± 11.3
**CS/CEO-2**	−16.9 ± 1.3	0.58 ± 0.08	50.21 ± 7.58	71.1 ± 10.5
**CS/CEO-2.5**	−22.4 ± 1.8	0.63 ± 0.09	47.67 ± 6.94	70.3 ± 10.2
